# ﻿*Balanophora xinfeniae* (Balanophoraceae), a new species from Xizang, China

**DOI:** 10.3897/phytokeys.266.147400

**Published:** 2025-11-19

**Authors:** Chen-Long Fu, Jia-Ning Zhou, Wei-Hua Liao, Tong Zhang, Bo Xu, Meng Li

**Affiliations:** 1 Co-Innovation Center for the Sustainable Forestry in Southern China, College of life sciences, Nanjing Forestry University, Nanjing 210037, Jiangsu, China Nanjing Forestry University Nanjing China; 2 Central South Inventory and Planning Institute of National Forestry and Grassland Administration, Changsha, 410014, China Central South Inventory and Planning Institute of National Forestry and Grassland Administration Changsha China; 3 CAS Key Laboratory of Mountain Ecological Restoration and Bioresource Utilization & Ecological Restoration and Biodiversity Conservation Key Laboratory of Sichuan Province, Chengdu Institute of Biology, Chinese Academy of Sciences, Chengdu 610213, Sichuan, China Chengdu Institute of Biology, Chinese Academy of Sciences Chengdu China

**Keywords:** *Balanophora xinfeniae*, China, holoparasitic plants, phylogeny, taxonomy

## Abstract

*Balanophora xinfeniae* C.L.Fu, M.Li & B.Xu, a new species discovered in Xizang, China, is described and illustrated here. Molecular phylogenetic analyses and morphological comparisons strongly support *B. xinfeniae* as a new species within the genus *Balanophora*. The most distinctive characteristic of the new species is its dioecious sexual system, with male flowers having a 3-lobed perianth, opposite leaves with serrated tips, and yellow scapes. Although it shares morphological similarities with *B. henryi*, *B. xinfeniae* can be distinguished by its flat spheroid tubers without stellate lenticels, yellow scape, prominently serrated apical leaf margins, and nearly spherical or ovoid-ellipsoid female inflorescences.

## ﻿Introduction

*Balanophora* J. R. Forst. & G. Forst. is a genus of holoparasitic plants consisting of about 25 species distributed mainly in tropical Southeast and East Asia (Hansen 1972; Mutinda et al. 2024). In traditional medicine, species of *Balanophora* are often used to treat hemorrhoids, stomach aches, and liver ailments and can also be used as antipyretics (Teng et al. 2000; Li et al. 2006; Trakulsomboon et al. 2006; Mutinda et al. 2024). Regarding their lifestyle, *Balanophora* comprises highly specialized root holoparasites characterized by underground connections with their hosts. Over an extended period of adaptive evolution, these plants have experienced significant gene loss from their genomes (Su et al. 2019; Chen et al. 2023) and extensive morphological reduction, leaving only a limited number of traits available for taxonomic classification (Job and Bertel 2015; Yu et al. 2022).

Morphologically, *Balanophora* can be readily divided into two subgenera: subgenus Balanophora (4- to 6-merous male flowers) and subgenus Balania (3-merous male flowers), a result that is also supported by molecular systematics (Yu et al. 2022). However, resolving relationships among these subgenera at the species level continues to be a challenging task. DNA regions with high substitution rates provide effective tools to evaluate the monophyly of *Balanophora* species defined by morphological traits. Recent studies have improved our understanding of species boundaries within this genus by comparing classifications based on morphological characters with results from molecular phylogenetic analyses (Hsiao et al. 2010; Su et al. 2012; Yu et al. 2021). For instance, Hansen (1972) considered the morphological differences among several species as intraspecific variation, synonymizing them under *B. harlandii*. In contrast, a more recent study based primarily on molecular phylogenetic analysis provided support for recognizing three distinct taxa—*B. harlandii*, *B. henryi*, and *B. kawakamii*—with clades corresponding to these taxa being strongly supported by molecular data (Yu et al. 2022).

During our fieldwork from April to May 2024 in the forests near Gelin Village, Motuo County, Xizang (China), we discovered a *Balanophora* that differed from all previously described species reported in China, India, Nepal, and other countries. Through morphological observations and the construction of a phylogenetic tree using nuclear ribosomal internal transcribed spacer (ITS) sequences, we concluded that this taxon represents an undescribed species of *Balanophora*.

## ﻿Materials and methods

### ﻿Morphological study

Morphological observations were conducted based on living plants in the field. Measurements were performed manually with rulers in the field or with the software ImageJ in the laboratory using photographs. We restricted morphological comparisons to three closely related species. At the same time, all images of specimens potentially related to the new species from China, India, Nepal, and other countries deposited in the herbaria A, CDBI, CDCM, GAC, HHBG, IBSC, IBK, LBG, K, KUN, NAS, P, PE, SHM, and ZM were observed and studied (acronyms follow Thiers 2024). The terminology used in the morphological description follows the Flora of China (Huang and Murata 2003). Observations of flowers and claviform bodies were carried out using an Olympus SZX10 stereomicroscope.

### ﻿Phylogenetic study

DNA extractions, PCR procedures, and primers followed Yu et al. (2021). The ITS region of the new species was sequenced using the Sanger method. In addition, we downloaded ITS sequences of other *Balanophora* species available in the Nucleotide database of NCBI (https://www.ncbi.nlm.nih.gov). *Langsdorffia hypogaea* was selected as the outgroup for this study based on the results presented by Sanchez-Puerta et al. (2023). A total of 54 samples were used in this study, including multiple accessions for some species (Table 1). All sequences were aligned using MAFFT v7.526 following default parameters (Katoh et al. 2002; Katoh and Standley 2013), and the ends were manually trimmed. The model TPM3u+F+I+R2 for ML analysis and TPM3uf+I+G for BI analysis was selected based on the Bayesian Information Criterion (BIC) using jModelTest v2.1.10 (Darriba et al. 2012). Maximum likelihood (ML) analyses were performed using IQ-TREE v2.3.6 with 10,000 ultrafast bootstrap approximations (Minh et al. 2020). A Bayesian inference (BI) analysis was performed using MrBayes v3.2.7 (Ronquist et al. 2012). A Markov chain Monte Carlo analysis was conducted for 20,000,000 generations with one cold and three hot chains, each starting from a random tree and sampled every 1,000 generations. The first 25% of the trees were discarded as burn-in, and the remaining trees were used to construct majority-rule consensus trees. The final trees obtained from the ML and BI analyses were visualized using FigTree v1.4.2 (Rambaut 2009).

**Table 1.** Taxa used in the phylogenetic analyses with their GenBank accession numbers.

Species	Accession number	Species	Accession number
*B. appressa*	MW414662	*B. laxiflora*	JN392888
*B. appressa*	MW414670	*B. laxiflora*	JN392889
*B. flava*	OM103570	*B. laxiflora*	MW414661
*B. flava*	OM103567	*B. laxiflora*	MW414659
*B. fungosa*	JN392881	*B. laxiflora*	JN392890
*B. harlandii*	OM103582	*B. laxiflora*	JN392891
*B. harlandii*	OM103583	*B. laxiflora*	JN392892
*B. harlandii*	OM103585	*B. laxiflora*	JN392893
*B. henryi*	OM103573	*B. parajaponica*	MT482093
*B. henryi*	OM103574	*B. parajaponica*	MT498081
*B. henryi*	OM103575	*B. parajaponica*	MW414666
*B. henryi*	OM103576	*B. parajaponica*	MW414667
*B. henryi*	OM103577	*B. reflexa*	EU598798
*B. indica*	JN392880	*B. reflexa*	KT763380
*B. involucrata*	OM103568	*B. subcupularis*	OM103584
*B. involucrata*	OM103569	*B. subcupularis*	OM418783
*B. involucrata*	OM103571	*B. subcupularis*	OM418784
*B. involucrata*	OM103572	*B. tobiracola*	JN392901
*B. japonica*	JN392882	*B. tobiracola*	OM103578
*B. japonica*	MW414664	***B. xinfeniae***	**PQ640032**
*B. japonica*	MW414665	***B. xinfeniae***	**PQ640033**
*B. kawakamii*	JN392900	*B. yakushimensis*	JN392894
*B. kawakamii*	OM103579	*B. yakushimensis*	MW414660
*B. kawakamii*	OM103580	*B. yakushimensis*	MW414663
*B. kawakamii*	OM103581	*B. yakushimensis*	MW414668
*B. latisepala*	OM418779	*B. yakushimensis*	MW414669
*B. latisepala*	OP120928	*Langsdorffia hypogaea*	KT709672

## ﻿Results and discussion

The circumscription of *B. flava*, *B. fargesii*, and *B. involucrata* remains a persistent taxonomic contention. Geng (2016) proposed that *B. fargesii* and *B. involucrata* are sister groups with distinct clades based on ITS sequences. In contrast, Lidén (2019) suggested that *B. fargesii* is synonymous with *B. involucrata*, while Yu et al. (2022) posited that *B. flava* and *B. fargesii* are synonymous with *B. involucrata*. In this study, these species are considered part of the *B. involucrata* complex, and we are not addressing taxonomic divisions within this group here. Instead, for clarity and to facilitate morphological comparisons, we refer to the dioecious individuals within the complex as *B. flava* and the monoecious individuals as *B. involucrata*.

**Figure 1.**
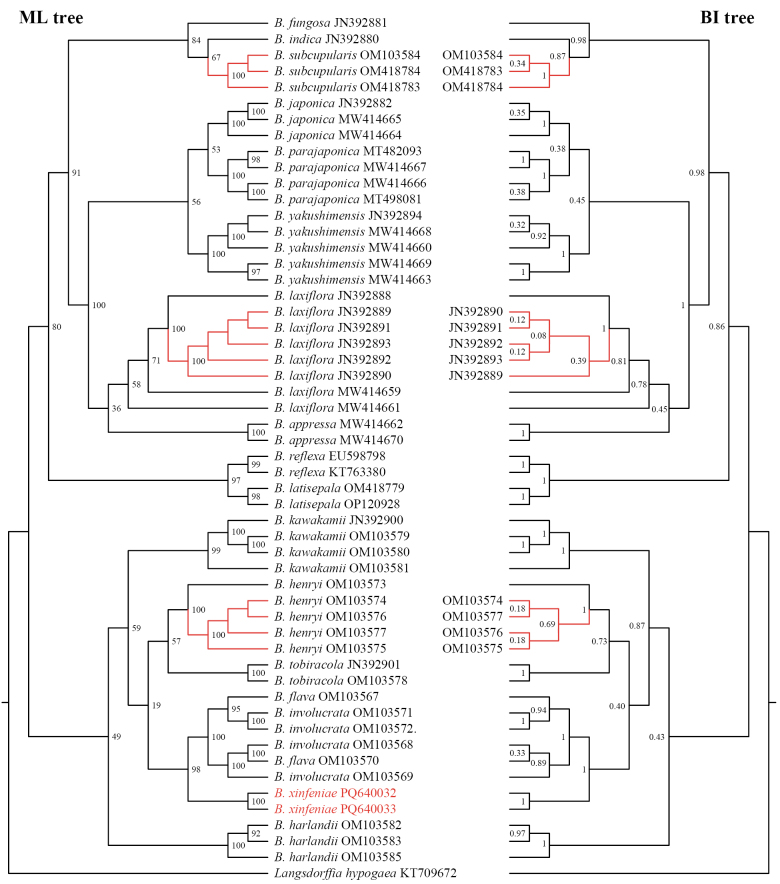
The cladogram of *Balanophora* constructed using maximum likelihood (left) and Bayesian inference (right) based on ITS sequences. Numbers at nodes indicate posterior probabilities (PP) or bootstrap percentages (BS). Branches highlighted in red indicate topological incongruence between the two trees. Labels for concordant topologies are not displayed on the BI tree.

The topologies of the phylogenetic trees constructed by the ML and BI methods are highly consistent (Suppl. materials 1, 2). Our results showed that two samples of the new species were well resolved as a distinct clade, which is the sister group to the *B. involucrata* complex (BS = 1; PP = 98%) (Fig. 1).

**Figure 2.**
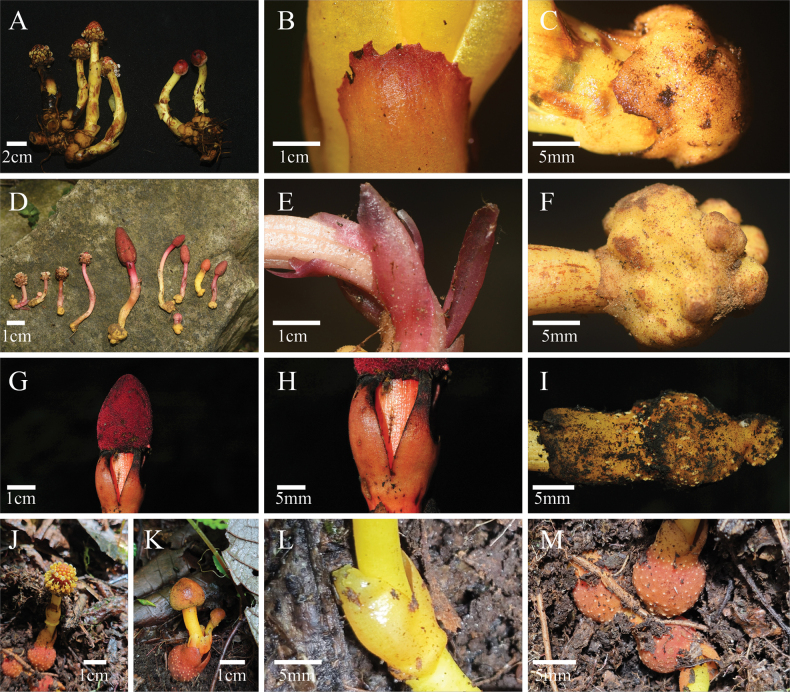
Morphology of *Balanophora xinfeniae* (**A–C**), *B. henryi* (**D–F**), *B. involucrata* (**G–I**), and *B. flava* (**J–M**). **A, D, G, J, K.** Male and female individuals; **B, E, H, L.** Leaves; **C, F, I, M.** Tubers. Photos by Meng Li and Wei-Hua Liao.

The new species described in this study is morphologically most similar to *B. henryi*, along with the closely related species *B. involucrata* and *B. flava*. In the phylogeny, the new species was placed in a clade containing these three species (Fig. 1). We therefore focused our comparison of morphological characters on these four species (Table 2; Fig. 2). The most distinctive characteristics of the new species are its dioecious sexual system (vs. monoecious in *B. involucrata*), opposite leaves (vs. connate into a single sheath-like whorl in *B. flava* and *B. involucrata*) with serrated margins (vs. crenate in *B. flava* and *B. involucrata* and entire in *B. henryi*), and yellow scapes (vs. red in *B. involucrata* and red or red to yellow in *B. henryi*).

**Table 2.** Comparison of morphological features among four related species of *Balanophora*.

	*B. xinfeniae*	*B. henryi*	*B. involucrata*	*B. flava*
Reproduction	Dioecious	Dioecious	Monoecious	Dioecious
Tuber branching shape	Flat spheroid or subglobose	Irregularly spherical or oblate	Urceolate, slightly elongated	Urceolate
Tuber surface	Densely covered with granular warts	Densely covered with granular warts	Densely covered with granular warts and yellowish stellate lenticels	Densely covered with granular warts and yellowish stellate lenticels
Color of scapes	Yellow	Red or red to yellow	Red	Yellow
Leaf arrangement	Opposite	Opposite	Connate into a single sheath-like whorl	Connate into a single sheath-like whorl
Leaves apex	Serrated	Entire	Crenate	Crenate
Shape of female inflorescence	Nearly spherical or ovoid-ellipsoid	Broadly ovoid	Ovoid or short ellipsoid	Ovoid or short ellipsoid
Flowering period	April to May	September to November	July to August	July to August

Based on the results of molecular and morphological analyses, we believe that there is sufficient evidence to support the validity of this taxon as distinctly different from *B. henryi*, *B. involucrata*, and *B. flava*. Therefore, we recommend that it be treated as a new species.

### ﻿Taxonomic treatment

#### ﻿ Balanophora xinfeniae

Taxon classificationPlantaeSantalalesBalanophoraceae

C.L.Fu, M.Li & B.Xu sp. nov.

8D91C8DC-9D08-572F-9539-ED2FD52BCD79

urn:lsid:ipni.org:names:77372106-1

Figs 3, 4

##### Type.

China • Xizang Autonomous Region: Motuo County (Mêdog), Gelin Village, 29.210161, 95.191856, alt. 1650 m, 29 Apr 2024 (fl.), *Meng Li, C.L. Fu & J.N. Zhou YLZB11691-A* (holotype: CDBI! Barcode number: CDBI0298364, ♀; isotypes: NAS! Barcode number: NAS00708465; NF!).

**Figure 3.**
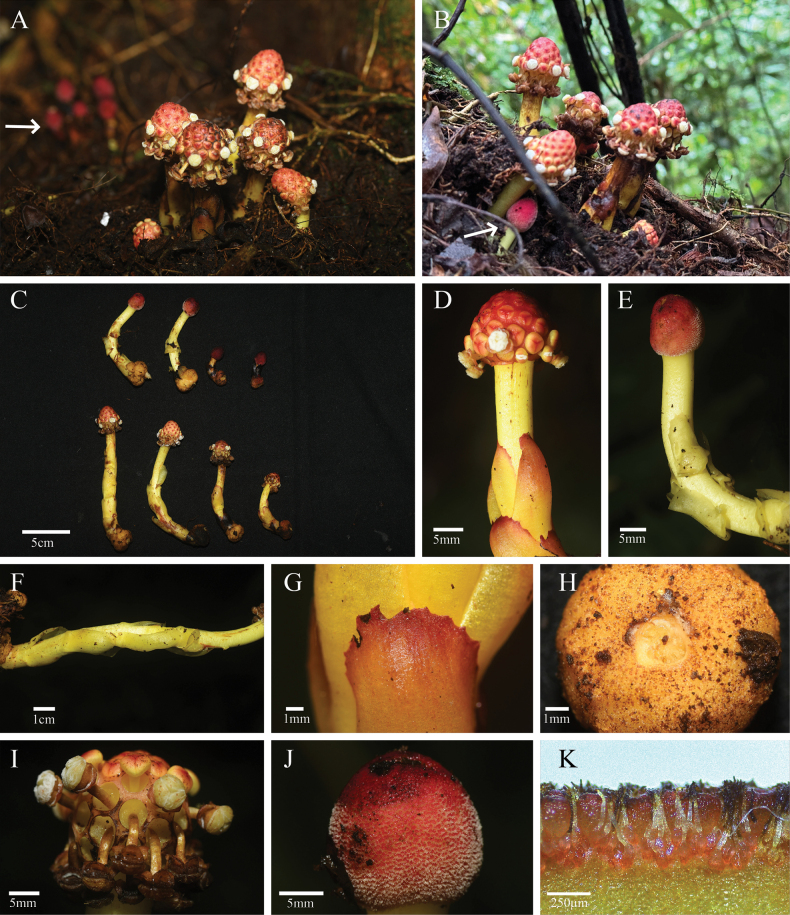
*Balanophora xinfeniae* C.L. Fu, M. Li & B. Xu. **A, B.** Habitats (both male and female; arrows point to female individuals); **C.** Female individual (upper) and male individual (lower); **D.** Male individual; **E.** Female individual; **F.** Leaves and scape; **G.** Leaves; **H.** Tuber; **I.** Male inflorescence; **J.** Female inflorescence; **K.** Female flower and claviform body. Photos by Meng Li and Chen-Long Fu.

##### Diagnosis.

*Balanophora xinfeniae* is morphologically similar to *B. henryi*, but the two species can be distinguished by several features. The tuber branching shape of *B. xinfeniae* is characterized as flat spheroid or subglobose, whereas that of *B. henryi* is irregularly spherical or oblate. The scapes of *B. xinfeniae* are yellow, in contrast to the red or red-to-yellow scapes of *B. henryi*. In *B. xinfeniae*, the apical margins of the leaves are prominently serrate, whereas those of *B. henryi* are entire. The female inflorescence of *B. xinfeniae* is nearly spherical or ovoid-ellipsoid, while that of *B. henryi* is broadly ovoid. The flowering period of *B. xinfeniae* is from April to May, compared to September to November for *B. henryi* (Table 2; Fig. 2).

**Figure 4.**
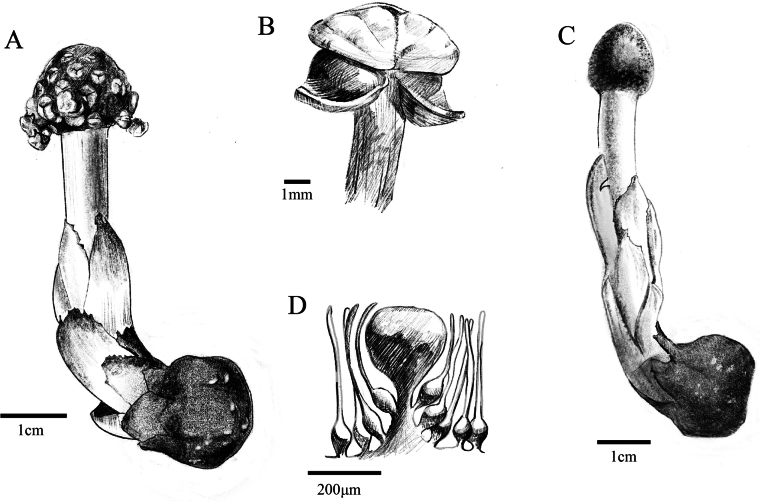
Line illustration of *Balanophora xinfeniae*. **A.** Male individual; **B.** Male flower; **C.** Female individual; **D.** Female flowers surrounding one claviform body. Drawn by Zi-Heng Yu based on YLZB11691-A and YLZB11691-B specimens stored in CDBI.

##### Description.

Plants dioecious. Tuber yellowish brown, branched or unbranched, surface scabrous, densely covered with granular warts; tuber branches flat spheroid or subglobose, 0.8–2.0 cm in diam. Scapes yellow, 2–12 × 0.5–1.2 cm. Leaves 6–10, yellow to reddish, opposite, scaly, oblong-ovate, apical margins prominently and irregularly serrate, with serrations arranged approximately parallel to the leaf margin, 1.2–3.5 × 0.3–1.8 cm. Male inflorescences subspheroid to ovoid-ellipsoid, 1.8–2.5 × 1.5–2.0 cm. Bracts truncate with expanded liplike margin, fused side by side into a hexagonal alveolus. Male flowers: pedicellate, inserted basally in alveolus, 3-merous, 2–4 mm in diam. Perianth lobes broadly deltoid. Synandria subdiscoid; anthers 3, transversely dehiscent. Female inflorescence nearly spherical or ovoid-ellipsoid. Claviform bodies obovoid, shortly stiped, apically truncate; cuticular ridges of apical cells labyrinth-like. Female flowers: both in the lower parts of the claviform bodies and on the main axis of the inflorescence.

##### Distribution and habitat.

**Figure 5.**
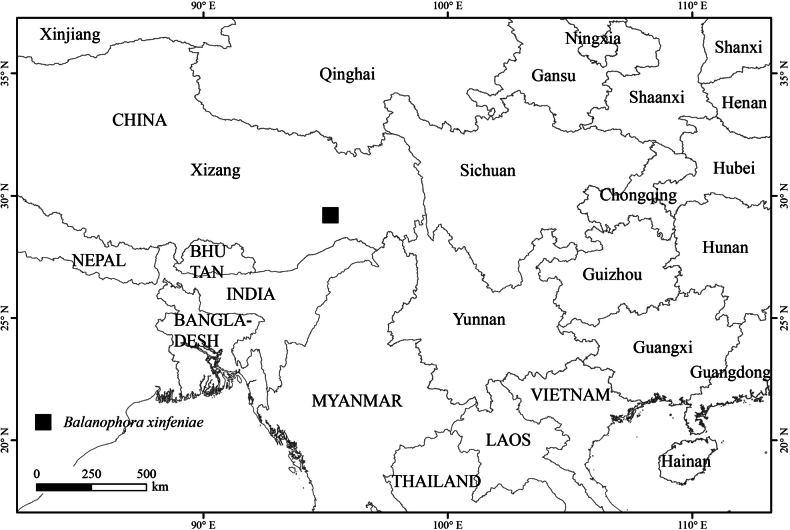
Distribution of *Balanophora xinfeniae* (Balanophoraceae).

The new species is currently known only from the type locality in Gelin Village, Motuo County, Xizang Autonomous Region (Fig. 5). It grows in shaded forest habitats on moist mountain slopes. The hosts are usually species of Fagaceae, such as *Quercus lamellosa* and *Castanopsis indica*.

##### Phenology.

The species has been observed flowering from April to May.

**Etymology.** The species epithet *xinfeniae* honors Prof. Xin-Fen Gao (former curator of the CDBI Herbarium), a distinguished botanist who made significant contributions to the taxonomy of seed plants, lycopods, and pteridophytes.

##### Vernacular name.

Chinese Mandarin: Xìn fēn shé gū (信芬蛇菰).

##### Additional specimens examined.

China • Xizang Autonomous Region: Motuo County, Gelin Village, 29.210161°N, 95.191856°E, alt. 1650 m, 29 Apr 2024 (fl.), *Meng Li, C.L. Fu & J.N. Zhou YLZB11691-B* (paratype: CDBI! Barcode number: CDBI0298363, ♂; NAS! Barcode number: NAS00708510, ♂; NF!, ♂); • Gelin Village, 29.220615°N, 95.189094°E, alt. 1602 m, 1 May 2024 (fl.), *Meng Li MD2405013-B* (paratype: CDBI! Barcode number: CDBI0298362, ♂); • Gelin Village, alt. 1700 m, 24 May 1983 (fl.), *B.S. Li, S.Z. Cheng & Z.C. Nie 03761* (PE! Barcode number: PE01491372; PE01801408); • Beibeng Township, alt. 1650 m, 11 May 1983 (fl.), *S.Z. Cheng, Z.C. Nie & B.S. Li 04844* (PE! Barcode number: PE01491371).

### ﻿Key to the new species of *Balanophora* and three morphologically similar taxa

**Table:** 

1	Leaves connate into a single sheath-like whorl	**2**
–	Leaves opposite	**3**
2	Plants monoecious, male flowers clustered basally on inflorescence	***Balanophora involucrata***
–	Plants dioecious	***Balanophora flava***
3	Scapes yellow, apical leaf margins prominently and irregularly serrate, female inflorescence nearly spherical or ovoid-ellipsoid	***Balanophora xinfeniae***
–	Scapes red or red to yellow, leaf margins entire, female inflorescence broadly ovoid	***Balanophora henryi***

## Supplementary Material

XML Treatment for Balanophora xinfeniae

## ﻿Additional information

### Use of AI

No use of AI was reported.

### Author ORCIDs

Chen-Long Fu https://orcid.org/0000-0003-3752-461X

Jia-Ning Zhou https://orcid.org/0009-0005-4280-4706

Wei-Hua Liao https://orcid.org/0009-0004-3504-3155

Tong Zhang https://orcid.org/0009-0009-1492-585X

Bo Xu https://orcid.org/0000-0003-3507-9321

Meng Li https://orcid.org/0000-0003-2085-3388

### Data availability

All of the data that support the findings of this study are available in the main text or Supplementary Information.

## Supplementary materials

Supplementary material 1 Multiple-sequence alignment in FASTA format Data type fasta https://binary.pensoft.net/file/1469585This dataset is made available under the Open Database License (http://opendatacommons.org/licenses/odbl/1.0/). The Open Database License (ODbL) is a license agreement intended to allow users to freely share, modify, and use this Dataset while maintaining this same freedom for others, provided that the original source and author(s) are credited.Chen-Long Fu, Jia-Ning Zhou, Wei-Hua Liao, Tong Zhang, Bo Xu, Meng Li

Supplementary material 2 Multiple-sequence alignment in NEX format Data type nex https://binary.pensoft.net/file/1469586This dataset is made available under the Open Database License (http://opendatacommons.org/licenses/odbl/1.0/). The Open Database License (ODbL) is a license agreement intended to allow users to freely share, modify, and use this Dataset while maintaining this same freedom for others, provided that the original source and author(s) are credited.Chen-Long Fu, Jia-Ning Zhou, Wei-Hua Liao, Tong Zhang, Bo Xu, Meng Li
